# Multi-target mechanisms and therapeutic potential of Dahuang Zhechong pill in lower extremity peripheral artery disease: a structured narrative review

**DOI:** 10.3389/fphar.2026.1766592

**Published:** 2026-05-26

**Authors:** Yue Teng, Youzhi Zhang, Bochuan Lv, Jintao Xu

**Affiliations:** 1 The First Clinical Medical College, Heilongjiang University Of Chinese Medicine, Harbin, China; 2 Interventional Department, First Affiliated Hospital of Heilongjiang University of Chinese Medicine, Harbin, China

**Keywords:** clinical application, Dahuang Zhechong Pill, lower extremity peripheral artery disease, multi-target mechanisms, traditional Chinese medicine

## Abstract

Lower extremity peripheral artery disease (PAD) is a chronic ischemic disorder primarily driven by atherosclerosis (AS) and characterized by endothelial injury, lipid deposition, inflammation, oxidative stress, and hemorheological abnormalities. From the perspective of traditional Chinese medicine (TCM), its pathogenesis is commonly interpreted within the concept of “*Tuoju*”, in which blood stasis obstructs the collaterals/meridians and may coexist with qi deficiency, blood heat, or phlegm-dampness. Dahuang Zhechong Pill (DHZCP), first recorded in the *Jingui Yaolue*, is traditionally prescribed to remove blood stasis, clear heat, and resolve masses, while also supporting vital qi and nourishing yin, which conceptually aligns with TCM pattern differentiation in PAD. Preclinical studies and limited clinical reports suggest that DHZCP may be associated with multi-domain effects relevant to PAD pathophysiology, including endothelial-related readouts, lipid metabolism, inflammation, oxidative stress, and hemorheological parameters. Clinical studies (mostly small-sample trials) indicate that DHZCP may be associated with improvements in ankle–brachial index (ABI), lipid profiles, blood viscosity, and microcirculatory perfusion, particularly when used as an adjunct to acupuncture, blood-activating prescriptions, or conventional therapies, with generally favorable short-term safety signals. This review summarizes the traditional rationale and modern evidence on DHZCP for PAD, critically appraises the current evidence base and its limitations, and proposes priorities for future research to support standardized clinical use and potential international application. This article is a structured narrative review based on a predefined literature search and study selection strategy. *In silico* findings (e.g., network approaches and molecular docking) are treated as hypothesis-generating evidence and are clearly distinguished from *in vitro*/*in vivo* and clinical studies.

## 1 Introduction

Lower extremity peripheral artery disease (PAD) is a common atherosclerotic vascular disorder primarily affecting the lower extremities and represents a major cause of morbidity worldwide (Takahara, 2021). It is characterized by progressive intimal thickening and atherosclerotic plaque formation, leading to arterial stenosis or occlusion and impaired distal perfusion (Wu Y et al., 2022). Sharing a common atherosclerotic basis with coronary artery disease and ischemic stroke, PAD is widely regarded as a peripheral vascular manifestation of systemic AS (Criqui et al., 2021). According to contemporary clinical guidelines, the occurrence and progression of PAD are strongly associated with modifiable risk factors, including smoking, diabetes mellitus, hypertension, and dyslipidemia, and the disease predominantly affects middle-aged and older adults (Gornik et al., 2024). Recent epidemiological data indicate a growing disease burden in China and globally, reflected by increasing incidence, prevalence, and disability-adjusted life years (Hu Y et al., 2024; Yu et al., 2025). Although antiplatelet therapy, lipid-lowering therapy, endovascular intervention, and surgical revascularization can improve perfusion and alleviate symptoms, key challenges persist, including restenosis and target-lesion revascularization after intervention (Li et al., 2024). These limitations highlight the need for optimized secondary prevention and adjunct strategies that address the metabolic-inflammatory-thrombotic milieu underlying PAD progression.

From the perspective of traditional Chinese medicine (TCM), PAD is commonly interpreted in relation to patterns such as qi stagnation with blood stasis, damp-heat with stasis obstruction, and yin deficiency with blood stasis (Nature, 2017). Accordingly, treatment principles emphasize activating blood circulation, unblocking collaterals to relieve pain, and supporting qi while nourishing yin, reflecting a multi-target and multi-pathway regulatory approach (Zeng et al., 2020). Modern studies have suggested that TCM-based interventions may improve hemorheological parameters, enhance microcirculation and collateral formation, relieve lower-limb symptoms, and potentially slow disease progression (Zheng et al., 2020). Within this context, Dahuang Zhechong Pill (DHZCP), first recorded in the *Jingui Yaolue* by Zhang Zhongjing (Eastern Han Dynasty; chapter “On Blood Impediment and Deficiency-Taxation Syndromes”), is traditionally used to dispel blood stasis and unblock collaterals while supporting deficiency, and is indicated for syndromes characterized by deficiency-taxation and blood stasis obstructing the collaterals. Contemporary pharmacological studies have reported that DHZCP is associated with blood-activating effects and may exhibit anti-inflammatory, antioxidant, lipid-modulating, antithrombotic, and hemorheology-improving activities (Zhou et al., 2020; Chen et al., 2022; Shi et al., 2025).

In recent years, network-based analyses, molecular docking, and multi-omics analyses have been used to generate hypotheses regarding candidate targets and pathways of DHZCP (Zhang et al., 2023). However, these *in silico* analyses remain preliminary and require functional validation in PAD-relevant experimental models and/or clinical studies (Yan et al., 2024; Diao et al., 2026). Accordingly, findings derived from in silico analyses can a best be interpreted as hypothesis-generating and cannot a mechanistic proof. Current experimental studies suggest that DHZCP may be associated with modulation of inflammation- and endothelial-related pathways, including NF-κB and p38 MAPK, whereas evidence for other computationally predicted pathways remains limited and context-dependent (He Y. et al., 2024). Animal and cellular experiments have reported directionally consistent changes in inflammatory cytokines (e.g., TNF-α, IL-1β, IL-6), oxidative-stress-related readouts, and endothelial functional indicators after DHZCP-related interventions (He et al., 2024a). Nevertheless, translation to PAD remains constrained in parts of the literature because many studies use non-PAD models and incompletely report dosing/exposure and control conditions. In addition, DHZCP has been proposed to influence inflammation-coagulation-endothelium interactions (Shi et al., 2025; Tung et al., 2023). Overall, the available evidence supports biological plausibility for multi-pathway regulation, but causal validation in PAD-relevant models remains limited.

Despite these signals, the current mechanistic understanding of DHZCP in PAD remains incomplete. First, an integrated synthesis that links material basis (intervention form), candidate metabolites, target/pathway modulation, and PAD-relevant functional outcomes is still lacking, as many studies focus on single targets or isolated pathways without establishing a coherent evidence chain across prediction, experimental validation, and clinical translation. Second, correlations between putative bioactive plant metabolites and specific signaling pathways remain insufficiently characterized in PAD contexts. For example, direct evidence on *in vivo* pharmacokinetics/exposure and mechanistic roles of metabolites such as emodin and paeoniflorin in PAD-related processes remains scarce. Third, a framework that systematically connects network-based predictions, omics-level validation, *in vivo* PAD-relevant evidence, and clinical evaluation is still underdeveloped. As a result, many reported associations cannot yet be interpreted as causal relationships between metabolites, targets, and therapeutic outcomes.

PAD is closely associated with chronic metabolic disorders, including diabetes mellitus, dyslipidemia, and hypertension. Its pathogenesis is characterized by complex interactions among metabolic dysregulation, persistent inflammatory activation, endothelial dysfunction, and prothrombotic hemorheological alterations. Within this multifactorial framework, therapeutic strategies that target interconnected pathological networks rather than single pathways may offer distinct advantages. From this perspective, TCM interventions, with their holistic and systems-oriented characteristics, warrant further investigation. DHZCP may exert synergistic therapeutic effects through multi-component, multi-target, and multi-pathway mechanisms, potentially modulating shared pathological processes underlying metabolic disorders and AS. This review summarizes evidence on DHZCP in PAD from four dimensions: TCM pathogenesis, formula composition, modern pharmacological mechanisms, and clinical application, with an explicit critical appraisal of evidence strength, limitations, and priorities for future research, thereby providing a rationale for integrative strategies in PAD management.

## 2 Methods

### 2.1 Literature search and study selection

A structured literature search was conducted from database inception to 30 July 2025 in PubMed, Embase, Web of Science Core Collection, Cochrane Library, China National Knowledge Infrastructure (CNKI), Wanfang database, China Biology Medicine disc (CBMdisc), China Science and Technology Journal Database (CSTJD), and China Biomedical Literature Database (SinoMed). Search terms covering both the intervention and condition were adapted to each database. A representative PubMed query was used as follows: (“Dahuang Zhechong” OR “Dahuangzhechong” OR “Da Huang Zhe Chong” OR “大黄蛰虫” OR “大黄䗪虫” OR “大黄蛰虫丸” OR “大黄䗪虫丸”) AND (“arteriosclerosis obliterans” OR “lower extremity arteriosclerosis obliterans” OR “peripheral artery disease” OR “Lower extremity peripheral artery disease” OR “PAD” OR “peripheral arterial disease” OR PAD OR “lower limb ischemia”). Searches were performed in title/abstract fields where applicable, and controlled vocabulary terms (e.g., MeSH/Emtree) were combined with free-text terms when available. Corresponding Chinese keywords were used in Chinese databases (e.g., “大黄蛰虫丸/大黄䗪虫丸”, “下肢动脉硬化闭塞症”, “外周/周围动脉疾病”). A representative CNKI query was: (“大黄蛰虫丸” OR “大黄䗪虫丸 OR “大黄蛰虫” OR “大黄䗪虫”) AND (“下肢动脉硬化闭塞症” OR “外周动脉疾病” OR “周围动脉疾病” OR “下肢缺血”). In addition, reference lists of eligible articles and relevant reviews were manually screened to identify further records.

Eligible evidence was defined as (i) experimental studies (*in silico*, *in vitro*, or *in vivo*) in which DHZCP, DHZCP-derived preparations (e.g., extracts/serum pharmacology), or plausible bioactive metabolites attributable to DHZCP ingredients were investigated with outcomes relevant to PAD-related processes (endothelial dysfunction, inflammation, oxidative stress, lipid metabolism, thrombosis/hemorheology); and (ii) clinical studies in PAD or closely related lower-limb macrovascular disease populations in which perfusion/vascular outcomes (e.g., ABI, PWV, Doppler parameters), biomarkers, symptoms, and/or safety were reported. Duplicated reports, conference abstracts without full text, studies with unclear intervention composition, and articles not relevant to DHZCP or PAD were excluded.

Records were deduplicated using reference management software. Titles/abstracts and full texts were screened by at least two authors, and disagreements were resolved by discussion. Key items were extracted, including study design, model/population, intervention material (commercial pill vs. laboratory preparation/extract vs. serum pharmacology vs. isolated metabolites, when reported), dose/concentration (if reported), comparators/controls, duration, outcomes, and major limitations. *In silico* findings (network approaches/molecular docking) were treated as hypothesis-generating unless functional validation was provided, and the evidence was synthesized narratively with explicit distinction among *in silico*, *in vitro*, *in vivo*, and clinical studies. Dose/concentration ranges, duration, and the use of positive/negative controls were extracted when reported; missing information was recorded as not reported (NR).

To assess whether new eligible evidence had emerged since the initial search, an updated search was performed up to 31 January 2026. After screening against the predefined eligibility criteria, no additional eligible mechanistic or clinical studies directly evaluating DHZCP for PAD were identified beyond those included.

### 2.2 Critical appraisal and evidence interpretation

A qualitative critical appraisal was performed for the included studies. For experimental studies, key items were assessed, including model relevance to PAD, intervention characterization (commercial product/extract/serum pharmacology/metabolite), dose/concentration plausibility, presence of appropriate controls, and completeness of reporting. For clinical studies, trial design features were considered, including randomization/blinding (when reported), baseline comparability, outcome selection (surrogate vs. hard endpoints), follow-up duration, and safety reporting. The overall certainty of evidence was interpreted conservatively, and *in silico* findings were treated as hypothesis-generating only.

## 3 TCM framework and formula rationale

### 3.1 *Tuoju* and PAD: classical descriptions and modern interpretation

In the TCM system of disease and syndrome classification, PAD is most commonly categorized under the condition known as *Tuoju* (Li Q et al., 2020). Ancient physicians described the core pathology of *Tuoju* as “obstruction of the blood vessels and insufficiency of qi and blood,” which is conceptually consistent with ischemia-induced tissue injury caused by impaired limb perfusion (Feng et al., 2024). Among ancient medical texts, the *Wai Ke Zheng Zong* (He and Wang, 2015), particularly the chapter “On *Tuoju*,” provides a representative description. It characterizes *Tuoju* as “external decay with internal deterioration” and attributes its development to factors such as excessive intake of greasy foods, overexertion, excessive sexual activity, and inappropriate use of warming and tonic botanical drugs. These factors were thought to impair organ function and deplete qi and blood, leading to internal heat, disruption of yin-yang balance, vascular obstruction, and distal tissue necrosis. The *Zhu Bing Yuan Hou Lun* (Li and Zhao, 2016) further attributes *Tuoju* to invasion by cold-damp pathogens combined with deficiency of vital qi, which, over time, leads to skin desiccation, darkening of lesions, and necrosis, highlighting the role of “deficiency with cold-induced stagnation.” The *Yi Zong Jin Jian* proposes that qi-blood deficiency is fundamental, whereas exogenous factors (wind, cold, dampness, heat) are secondary; pain arises when blood becomes stagnant, reflecting a “root deficiency with superficial excess” pattern (Huang, 2003). The *Zheng Zhi Zhun Sheng* similarly notes that qi-blood deficiency predisposes to cold-damp invasion, resulting in prolonged obstruction and eventual gangrene (Chen, 2014). The *Yang Yi Da Quan* describes onset from cold invasion of the feet with stagnation of blood flow; if prolonged, cold may transform into heat and generate toxic congestion, suggesting a progression from excess cold to internal heat-toxin accumulation (Yu et al., 2018).

Overall, classical TCM texts consistently emphasize a multi-factor pathogenesis involving organ dysfunction, qi-blood deficiency, cold-damp invasion, and vascular obstruction in the evolution of *Tuoju* (Xiong et al., 2021). Taken together, these descriptions provide a traditional theoretical framework that can be used to interpret PAD in terms of deficiency-stasis interactions and cold/heat transformation, which in turn informs syndrome differentiation and treatment strategies.

### 3.2 TCM pathogenesis and treatment principles for PAD

Building on the classical understanding of the pathogenesis of *Tuoju*, modern TCM has developed a more systematic interpretation of PAD, emphasizing organ function, qi-blood circulation, and collateral obstruction (Wang et al., 1999). Compared with the traditional concept of *Tuoju*, PAD is described as a more complex condition characterized by the dynamic interplay of “root deficiency and superficial excess.” Among these, spleen deficiency is commonly regarded as the fundamental basis, often related to irregular diet, chronic illness, or impaired transport and transformation; these factors weaken the generation of qi and blood and reduce the capacity to propel circulation. Qi stagnation is considered an important aggravating factor that impedes blood flow and contributes to stasis (Lu et al., 2024). Blood stasis, in turn, is regarded as a core pathogenic factor throughout the disease course, contributing to vascular obstruction and trophic insufficiency of the extremities, which is broadly consistent with modern concepts such as arterial narrowing, microcirculatory impairment, and tissue ischemia (Song and Guo, 2022). In some patients, excessive intake of greasy foods or metabolic imbalance is thought to generate internal damp-heat, leading to the accumulation of dampness, heat, phlegm, and stasis, thereby worsening obstruction (Xing et al., 2023). In advanced stages, deficiency of the liver and kidneys and depletion of yin fluids may occur, resulting in malnourishment of tendons and vessels and manifesting as chronic ischemic symptoms such as numbness, cold pain, and skin atrophy.

Taken together, the TCM pathogenesis of PAD can be summarized as a deficiency-based disorder (particularly spleen deficiency and qi deficiency) with blood stasis obstructing the collaterals as a prominent manifestation, often complicated by damp-heat and yin deficiency. Based on this framework, treatment principles emphasize invigorating blood circulation, unblocking collaterals to relieve pain, and supporting qi while nourishing yin, with particular attention to “removing stasis to generate new,” aiming to improve local perfusion and promote tissue repair. Within this theoretical context, DHZCP is frequently cited as a representative formula that combines stasis-resolving and deficiency-supporting strategies, providing a potential rationale for improving hemodynamics and alleviating ischemia in PAD (Cheng et al., 2024).

### 3.3 DHZCP: composition, formula logic, and material definition

**FIGURE 1:**
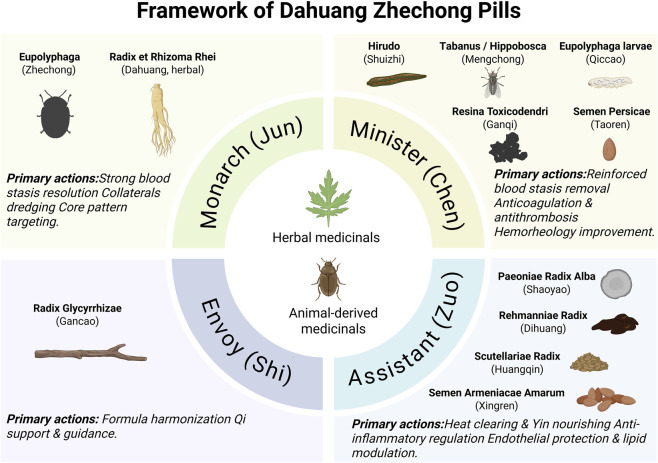
Schematic illustration of the Jun-Chen-Zuo-Shi (sovereign-minister-assistant-courier) hierarchy and formula composition of Dahuang Zhechong Pill (DHZCP).

DHZCP was first recorded in the *Jingui Yaolue*, in the chapter “Patterns and Treatments of Blood Impediment and Deficiency-Taxation Diseases.” It is one of Zhang Zhongjing’s classical formulas designed to address syndromes involving internal blood stasis, such as “dry blood” and “skin thickening and nail discoloration” (*Jifu Jia Cuo*). The prescription consists of twelve medicinal materials: *Rhei Radix et Rhizoma* (Dahuang), *Scutellariae Radix* (Huangqin), *Glycyrrhizae Radix et Rhizoma* (Gancao), *Persicae Semen* (Taoren), *Armeniacae Semen Amarum* (Xingren), *Paeoniae Radix Alba* (Shaoyao), *Rehmanniae Radix* (Gandihuang), *Toxicodendri Resina* (Ganqi), *Tabanus* (Mengchong), *Hirudo* (Shuizhi), *Holotrichia* (Qicong), and *Eupolyphaga* (Zhechong). The formula follows a Jun-Chen-Zuo-Shi (sovereign-minister-assistant-courier) hierarchy, integrating stasis removal and collateral unblocking, heat clearing and mass resolving, and deficiency support with regeneration (Figure 1). In the formula, Dahuang and Zhechong serve as the sovereign drugs, working synergistically to dispel blood stasis and promote circulation, thereby targeting the central pathogenesis of “collateral obstruction due to stasis.” Taoren, Shuizhi, Mengchong, Qicong, and Ganqi act as minister drugs to reinforce blood-activating and stasis-resolving actions. Shaoyao, Gandihuang, Huangqin, and Xingren serve as assistant drugs to clear heat, moisten dryness, nourish yin and the collaterals, and alleviate pain, thereby helping to balance the formula and reduce the risk of injuring healthy qi. Gancao functions as the courier drug, harmonizing the formula and supporting middle-jiao qi, thereby strengthening overall coordination.

Definition of DHZCP and materials investigated. In this review, evidence related to “DHZCP” was categorized according to the intervention material used in primary studies, including (i) commercial DHZCP pill products, (ii) laboratory-prepared decoctions/extracts, (iii) DHZCP-containing serum (serum pharmacology), and (iv) isolated bioactive metabolites. Because these material forms differ in composition and exposure profiles, findings from one preparation were not considered directly interchangeable with those of another. Across the reviewed studies, the exact preparations were often insufficiently characterized, making it difficult to determine whether they were identical or directly comparable. Incomplete reporting of intervention characterization (e.g., product specification, preparation method, or batch consistency) therefore limits reproducibility and cross-study comparability.

Taxonomic verification. Taxonomic names of botanical drugs in DHZCP were verified against authoritative resources (POWO/MPNS and relevant pharmacopoeias), and full source species names (including authorities) and family names were provided where applicable (Table 1 (Kaplanski et al., 1998; Kazimírová et al., 2002; Wang et al., 2014; Wang and Du, 2016; Xu et al., 2016; Hyun et al., 2019; Zhao et al., 2020; Bhattamisra et al., 2021; Stompor-Gorący, 2021; Wang H et al., 2022; Li et al., 2023)). When primary studies did not report sufficient species- or product-level information, this was recorded as not reported (NR) and considered a limitation for reproducibility and cross-study comparability.

**TABLE 1:** Major medicinal materials of Dahuang Zhechong pill (DHZCP): source species, putative targets, evidence type, mechanisms, and reporting-quality considerations.

Role in formula	Drug name (Latin/English)	Type	Source species (authority) and family	Putative targets/Pathways	Evidence type	Mechanisms
Monarch	*Rhei Radix et Rhizoma* (Dahuang; Rhubarb root and rhizome) (Stompor-Gorący, 2021)	Botanical drug	*Rheum palmatum L./Rheum tanguticum (Maxim. ex Balf.) Maxim. ex Regel/Rheum officinale Baill.* [Polygonaceae] (pharmacopoeial sources; exact species NR in some studies)	NF-κB, VEGF, eNOS, MMP-2/9	*in vitro*/*in vivo*; some *in silico*	Reported inhibition of NF-κB-related signaling; modulation of VEGF-eNOS/NO axis; anti-inflammatory/antioxidant effects; lipid-related effects
	*Eupolyphaga* (Zhechong; ground beetle) (Wang Y et al., 2022)	Animal drug	*Eupolyphaga sinensis Walker*, 1868 [Corydiidae] (reported in TCM/Chinese literature; species reporting varies)	Thrombin, Fibrin, ICAM-1/VCAM-1	*in vitro*/*in vivo*; clinical (indirect)	Antithrombotic/hemorheology-related effects; reduction of adhesion molecules and microcirculatory improvement
Minister	*Persicae Semen* (Taoren; Peach kernel) (Wang et al., 2014)	Botanical drug	*Prunus persica* (L.) *Batsch* [Rosaceae]	TXA_2_ pathway, ADP-platelet pathway	*in vivo*/*in vitro*	Antiplatelet and microcirculation-related effects
	*Hirudo* (Shuizhi; Leech) (Kaplanski et al., 1998)	Animal drug	*Hirudo nipponia Whitman,* 1886 [Hirudinidae] (or Hirudo spp. [Hirudinidae]; species NR in some reports)	Thrombin, ICAM-1/VCAM-1	*in vivo*/*in vitro*; clinical (indirect)	Hirudin-mediated thrombin inhibition; anti-adhesion and endothelial inflammation attenuation
	*Tabanus* (Mengchong; Horsefly) (Kazimírová et al., 2002)	Animal drug	*Tabanus* spp. [Tabanidae] (species often NR)	Thrombin	*in vitro*/*in vivo*	Anticoagulant proteins; “blood-breaking” effect in TCM; antiplatelet/anti-adhesion signals reported in some studies
	*Holotrichia* (Qicong; Beetle larva) (Xu et al., 2016)	Animal drug	*Holotrichia* spp. [Scarabaeidae] (species often NR)	Fibrinolytic proteins	*in vitro*/*in vivo*	Fibrinolytic/antithrombotic activity; microcirculation-related effects
	*Toxicodendri Resina* (Ganqi; Lacquer resin)	Resin	*Toxicodendron vernicifluum (Stokes) F.A.Barkley* [Anacardiaceae] resin (commonly cited; exact material definition NR in many studies)	NR	NR/limited	Traditionally used for resolving stasis; modern vascular evidence limited/heterogeneous
Assistant	*Scutellariae Radix* (Huangqin; Baical skullcap root) (Zhao et al., 2020)	Botanical drug	*Scutellaria baicalensis* Georgi [Lamiaceae]	NLRP3, NF-κB, p38 MAPK	*in vitro*/*in vivo*; some *in silico*	Anti-inflammatory/antioxidant signals; inflammasome-related modulation
	*Paeoniae Radix alba* (Baishao; White peony root) (Li et al., 2023)	Botanical drug	*Paeonia lactiflora Pall.* [Paeoniaceae]	TLR4/NF-κB, VCAM-1/ICAM-1, MMP-2/9	*in vitro*/*in vivo*	Anti-inflammatory; reduced adhesion molecules; plaque-related matrix modulation
	*Rehmanniae Radix* (Dihuang; Rehmannia root) (Bhattamisra et al., 2021)	Botanical drug	*Rehmannia glutinosa (Gaertn.) DC.* [Orobanchaceae]	ROS/NOX4, TLR4/MyD88, eNOS	*in vitro*/*in vivo*	Oxidative stress reduction and endothelial protection signals (e.g., catalpol-related)
​	*Armeniacae Semen Amarum* (Xingren; Bitter apricot kernel) (Hyun et al., 2019)	Botanical drug	*Prunus armeniaca L.* [Rosaceae] (or related Prunus spp.; source species may vary by pharmacopeia)	TNF-α, IL-1β, IL-6, MMP-9	*in vitro*/*in vivo*	Anti-inflammatory signals (e.g., amygdalin-related); MMP-9 reduction in some models
Envoy	*Glycyrrhizae Radix et Rhizoma* (Gancao; Licorice root) (Wang and Du, 2016)	Botanical drug	*Glycyrrhiza uralensis Fisch. ex DC./Glycyrrhiza inflata Bat./Glycyrrhiza glabra L.* [Fabaceae] (pharmacopoeial sources; exact species NR in some studies)	NF-κB, MAPK, COX-2	*in vitro*/*in vivo*	Harmonizing role; anti-inflammatory and protective effects reported

Targets and mechanisms are summarized from heterogeneous primary studies. Across the included studies, it was often unclear whether the investigated DHZCP-related materials or preparations were identical or directly comparable. “NR” indicates not reported or unclear in the primary literature and signals insufficient reporting of source species, material definition, preparation method, product specification, or batch/manufacturer information where applicable. Therefore, these mechanistic claims should be interpreted as putative unless supported by exposure-aware and causally informative validation.

From a modern pharmacological perspective, the botanical drugs and insect-derived bioactive substances in DHZCP exhibit a functional division of labor (Table 1). Current evidence suggests that key botanical drugs (e.g., Dahuang, Huangqin, Gandihuang, Shaoyao) contain anthraquinones and flavonoids, including emodin and rhein (from Dahuang) and baicalin and baicalein (from Huangqin). These plant metabolites have been reported to influence inflammatory signaling (e.g., NF-κB and p38 MAPK) and modulate targets related to endothelial function and extracellular matrix remodeling (e.g., VEGF, eNOS, MMP-2/MMP-9), thereby contributing to anti-inflammatory and antioxidant effects and potentially improving lipid metabolism and endothelial function (Wu et al., 2014; Lin et al., 2012; Guo et al., 2022; Li and Ren, 2022; Wen et al., 2023). In contrast, insect-derived drugs (e.g., Shuizhi, Mengchong, Qicong) and resin materials are more frequently linked to anticoagulation/antithrombosis, inhibition of platelet aggregation, reduction of blood viscosity, and improvement of microcirculation. For example, hirudin, a key bioactive peptide derived from Shuizhi, has been validated as a thrombin inhibitor and has also been reported to attenuate endothelial activation by reducing ICAM-1/VCAM-1 upregulation in inflammatory settings (Kaplanski et al., 1998; Bao et al., 2012; Junren et al., 2021). Overall, the formula can be interpreted as combining botanical metabolites that are mainly linked to anti-inflammatory/antioxidant and endothelial-related effects with animal-derived bioactive substances that are mainly linked to antithrombotic and hemorheological regulation. Together, these actions are consistent with key pathological features of PAD, including inflammatory activation, endothelial dysfunction, lipid deposition, and thrombosis-prone hemorheological changes (Ye et al., 2022; Shi et al., 2024).

## 4 Evidence map of pharmacological mechanisms relevant to PAD

### 4.1 Evidence landscape: candidate metabolites, dose-exposure plausibility, and PAD-relevant validation gaps

To clarify the relationship between candidate metabolites, targets, and PAD-relevant effects, the mechanistic evidence was first synthesized at three levels: (i) frequently reported candidate metabolites, (ii) dose–response and exposure plausibility, and (iii) the extent of functional validation in PAD-relevant models. Across the reviewed literature, frequently discussed candidate bioactive plant metabolites that may contribute to DHZCP-related effects include emodin, rhein, baicalin/baicalein, paeoniflorin, and catalpol; these candidates have been repeatedly linked to endothelial, inflammatory, oxidative-stress, or lipid-related readouts in predominantly non-PAD experimental settings. However, none of these metabolites can currently be designated as definitive “core actives” for PAD because the available evidence remains predominantly associative rather than causal. In particular, clear dose–response relationships and minimal active concentrations were inconsistently reported, and pharmacokinetic exposure data connecting *in vitro* effective concentrations to *in vivo* achievable levels in PAD-relevant models were largely unavailable. Moreover, while network approaches and molecular docking (Li et al., 2022; Wang Y et al., 2022; Bisht et al., 2024) have generated multiple candidate targets/pathways (e.g., NF-κB, PI3K/Akt, p38 MAPK, NLRP3), functional validation in PAD-relevant limb ischemia/arterial stenosis models using inhibitors/agonists or genetic perturbation (knockdown/knockout) remained rare. Accordingly, aligned with the methodological caveats raised by Diao et al. (2026), *in silico* predictions were treated strictly as hypothesis-generating signals due to their inherent susceptibility to bias. Mechanistic interpretations were thus framed as biologically plausible hypotheses rather than confirmed causal mechanisms.

### 4.2 Endothelial function and vascular remodeling

Endothelial dysfunction is a critical early event in the onset and progression of PAD (Idei et al., 2011). Its development is closely associated with hemodynamic disturbances, smoking, hypercholesterolemia, hypertension, diabetes, and other risk factors (Pu et al., 2022). These conditions reduce NO production, trigger endothelial inflammatory activation, upregulate cell adhesion molecules, and impair angiogenic capacity, thereby promoting both the formation and destabilization of atherosclerotic plaques (Hayden, 2025). Against this background, numerous animal and cellular studies have suggested that DHZCP exerts multi-target effects, such as anti-inflammatory, antithrombotic, lipid-regulatory actions, and modulation of endothelial-related signaling pathways. DHZCP may alleviate endothelial injury and hemodynamic imbalance, though many data were obtained from non-PAD models and should be interpreted cautiously when extrapolated to lower-limb PAD (Shi et al., 2025).

At the whole-formula level, DHZCP was reported to upregulate VEGF and TGF-β, regulate the NOS system by reducing iNOS and increasing tNOS expression, and enhance NO production (Yan et al., 2025). These molecular changes are directionally consistent with improved vasomotor regulation and collateral development, but they remain surrogate readouts unless linked to PAD-specific perfusion endpoints. Moreover, DHZCP was reported to inhibit MMP-9 expression, thereby potentially reducing basement membrane degradation and extracellular matrix disruption (Dai et al., 2021). Representative studies in AS animal models reported that DHZCP diminished abnormally high expressions of molecules such as VEGF and MMP-9 in diseased vessels, suppressed inflammatory responses and extracellular matrix degradation, stabilized atherosclerotic plaques, and improved lipid profiles and inflammatory markers (Duan et al., 2022). Although these findings are based on AS models, they are likely to be pathophysiologically relevant to PAD, given that AS represents the fundamental pathological substrate of the disease. However, a critical gap remains, as direct validation in limb ischemia or arterial stenosis models is still lacking.

From a modern pharmacological perspective, multiple active plant metabolites in DHZCP have been reported to exert endothelial-protective effects, providing a mechanistic rationale for the formula’s overall efficacy. Among them, emodin was reported to significantly alleviate ox-LDL-induced endothelial cell injury by activating eNOS, enhancing NO production, and suppressing reactive oxygen species (ROS) and NF-κB activity, accompanied by reduced expression of ICAM-1 and MMP-9 (Wang et al., 2014; Li G et al., 2020). Paeoniflorin, another key metabolite, was reported to protect endothelial barrier structure and cell viability through dual mechanisms: upregulating the eNOS/NO pathway to improve endothelium-dependent vasodilation, and downregulating pro-inflammatory cytokines such as TNF-α and IL-1β, thereby mitigating oxidative stress (Ji et al., 2012; Wu J et al., 2022). Among the insect-derived bioactive substances, RGD/disintegrin-like molecules from Mengchong were reported to show anti-platelet aggregation and anti-adhesion properties (Ma et al., 2011). Taken together, the mechanistic narrative is internally coherent (endothelium-inflammation-thrombosis coupling), but the current evidence base still lacks exposure-aware and causally informative validation in PAD-specific models.

In summary, DHZCP may improve vascular endothelial structure and function through mechanisms including modulation of VEGF/NO signaling, inhibition of MMP-9 and adhesion molecule expression, and reduction of oxidative stress and inflammation. However, the overall certainty of evidence is constrained by heterogeneity of models (often not PAD-specific), incomplete reporting of dose/exposure in some studies, and limited causal validation using inhibitors or genetic perturbation. Moreover, PAD-specific limb ischemia/arterial stenosis models remain underrepresented in the published literature; therefore, much of the mechanistic evidence was derived from non-PAD AS or endothelial injury models.

Evidence level and limitations: Most evidence summarized in this section was derived from *in vitro* or *in vivo* AS/endothelial injury models rather than dedicated PAD models. *In silico* predictions were not treated as mechanistic proof unless supported by experimental validation. Across studies, key reporting gaps recurrently involved (i) incomplete dose/concentration ranges and a lack of minimal active exposure, (ii) limited use/reporting of positive and negative controls, and (iii) inconsistent characterization of intervention materials (commercial product vs. extract vs. serum pharmacology), all of which reduced mechanistic interpretability. Dose-response relationships and minimal active exposures were rarely established, because concentration/dose ranges and pharmacokinetic exposure information were frequently not reported (NR), limiting causal inference from surrogate endothelial readouts to PAD outcomes.

What the evidence shows/does not show: Preclinical studies reported directionally consistent changes in endothelial-related surrogate readouts (e.g., NO-related indices, adhesion molecules, MMP-9), but causal links to PAD-specific functional outcomes (limb perfusion, restenosis, amputation risk) were not established. Therefore, the current literature supports biological plausibility for endothelial protection but does not yet demonstrate causality or quantify clinically relevant effect sizes. In addition, most pathway claims were supported by associative biomarker changes, while inhibitor- or genetic perturbation-based validation in PAD-relevant models was rarely reported.

### 4.3 Lipid metabolism and lipid-related vascular risk

Lipid metabolism disorder is one of the pathological rationales in the onset and progression of PAD (Zhang J et al., 2025). Its mechanisms primarily involve elevated low-density lipoprotein cholesterol (LDL-C), reduced high-density lipoprotein cholesterol (HDL-C), and the oxidative modification of LDL to ox-LDL, which subsequently triggers a cascade of inflammatory responses, foam cell formation, and plaque deposition (Berberich and Hegele, 2012). Because lipid dysregulation both drives plaque progression and interacts with thrombosis-prone hemorheology (Klatt et al., 2018), it provides a second major mechanistic entry point for explaining DHZCP’s potential effects. DHZCP may improve lipid metabolic abnormalities through a multi-target, multi-pathway regulatory network, thereby potentially interrupting critical pathological processes of PAD. Animal experiments have reported that DHZCP reduced total cholesterol (TC), triglycerides (TG), and LDL-C levels, while increasing HDL-C content, although the translatability depends on model relevance, dosing plausibility, and material characterization (Shi et al., 2025). Reduced LDL may imply decreased ox-LDL generation, which could contribute to lowering lipid deposition in macrophages and foam cell formation, thereby potentially slowing early plaque development. In addition, studies using thrombosis and related disease models reported that DHZCP diminished plasma TXB_2_ levels and increased 6-keto-PGF_1_α, thereby correcting the TXA_2_/PGI_2_ imbalance and attenuating platelet activation and thrombogenicity (Wan et al., 2012). Importantly, these observations were largely derived from non-PAD models and should be interpreted as supportive signals rather than direct evidence for PAD clinical benefit.

Modern pharmacological studies have further proposed lipid-regulating mechanisms of several metabolites in DHZCP, mostly based on non-PAD metabolic or AS models. For example, emodin was reported to suppress lipid deposition, alleviate oxidative stress, and improve vascular endothelial function in high-fat diet and AS models. Rhein was reported to improve lipid metabolism and reduce hepatic lipid accumulation via the AMPK/ACC/SREBP1 pathway in obesity and non-alcoholic fatty liver disease models. It was suggested to protect against oxidative stress-related endothelial and vascular injury (Guo et al., 2022; Dai et al., 2025). Among the flavonoid metabolites of Huangqin, baicalin was reported in AS/hyperlipidemic models, to inhibit inflammatory signaling pathways such as p38 MAPK and NF-κB, accompanied by changes in lipid levels and lipid metabolism-related proteins, including SREBP-1c and PPARα (Wu J et al., 2022). Although hirudin (insect-derived bioactive peptide) from Shuizhi is not a traditional lipid-modulating agent, it has shown anticoagulant, antithrombotic, and hemorheology-modifying properties. Therefore, its contribution is more plausibly positioned as modifying thrombosis risk in the setting of plaque burden rather than directly lowering lipids (Junren et al., 2021).

Although most current evidence derives from AS models, the pathological processes of lipid metabolic disorder, foam cell formation, endothelial injury, and plaque progression are partially overlapping with those observed in PAD. In summary, DHZCP may exert synergistic effects related to lipid modulation, oxidative stress reduction, inflammation control, and hemodynamic improvement. Nevertheless, the strength of evidence for lipid-lowering efficacy in PAD is limited by the scarcity of well-controlled PAD-specific models and clinical trials with standardized endpoints and adequate follow-up.

Evidence level and limitations: Most evidence in this section comes from *in vivo* metabolic/AS models and mechanistic *in vitro* studies; PAD-specific validation and dose-exposure plausibility assessments were seldom reported. Across studies, incomplete dose/concentration reporting, limited positive/negative controls, and heterogeneous intervention materials (commercial product vs. extract vs. serum pharmacology) frequently constrained interpretability. Dose-response relationships and minimal active exposures were rarely established, and pharmacokinetic exposure data were seldom available; therefore, the magnitude of lipid effects and their translational relevance to PAD could not be quantified reliably.

What the evidence shows/does not show: Lipid improvements were repeatedly reported in non-PAD metabolic/AS models after DHZCP exposure, whereas evidence linking lipid changes to clinically meaningful PAD endpoints remains insufficient. Thus, the literature supports a lipid-modulating signal but does not yet establish whether such changes mediate improved limb perfusion or reduced limb events in PAD. Heterogeneous dosing, the limited exposure plausibility assessment, and model mismatch (non-limb ischemia) were common, highlighting the need for PAD-relevant models and standardized outcome reporting.

### 4.4 Inflammation-related pathways

The inflammatory response plays a central role throughout the progression of both AS and PAD, linking endothelial activation, leukocyte recruitment, and plaque evolution (Ajoolabady et al., 2024). Pro-inflammatory cytokines such as TNF-α, IL-1β, and IL-6 amplify immune cascade reactions and accelerate vascular wall inflammation. Meanwhile, persistent activation of signaling pathways such as NF-κB and the NLRP3 inflammasome promotes upregulation of adhesion molecules, polarization of macrophages toward the M1 phenotype, and intensification of localized inflammation (Chen and Li, 2024). Given this centrality, anti-inflammatory activity is frequently invoked as a key mechanistic rationale for DHZCP in vascular disease. Current studies suggest that DHZCP, through a multi-metabolite, multi-target profile, may suppress inflammatory responses and modulate the vascular inflammatory microenvironment. However, the extent of causal validation varies markedly across models and study designs.

Animal and cellular experiments have reported that DHZCP simultaneously regulates multiple inflammation-related pathways (He X. et al., 2024), with a consistent trend toward downregulating TNF-α, IL-1β, and IL-6 and inhibiting NF-κB activation, thereby potentially alleviating vascular inflammation and intimal thickening. Specifically, DHZCP was reported to reduce p-p65/p65 and p-IκBα/IκBα ratios in vascular tissues and decrease inflammatory mediator release, with concomitant suppression of plaque-associated injury signals in some models (Zhou et al., 2020). Other findings suggested inhibition of the IL-1β/NF-κB/NLRP3 axis, reduced inflammatory cell infiltration, and improved vascular remodeling (Hu Q et al., 2024). Additional evidence suggests that modulation of the PAF/LP-PLA2 signaling axis attenuates platelet activation and thrombosis, while mitigating inflammation-associated hemodynamic disturbances (Shi et al., 2025). Nevertheless, many of these studies were conducted in non-PAD settings, and inhibitor-based or genetic perturbation strategies were rarely applied to establish causality.

Consistent with whole-formula observations, mechanistic signals have also been proposed for individual metabolites. Baicalin and baicalein were reported to inhibit NF-κB and p38 MAPK signaling and reduce cytokine production and adhesion molecule expression (Si and Lai, 2024). Catalpol, a key plant metabolite in Gandihuang, was reported to attenuate inflammation-related signaling (e.g., TLR4/NF-κB) and oxidative stress in relevant models (Chen et al., 2019). For animal-derived materials, bioactive peptides rather than “metabolites” are typically implicated; for example, bdellin-like peptides from Shuizhi were suggested to inhibit protease activity and inflammatory mediator release (Chen et al., 2019). Polypeptides from Qicong were reported to reduce iNOS and TNF-α and influence macrophage polarization balance (Kwon et al., 2023). Polyphenols in Ganqi (e.g., gallic acid) were reported to suppress COX-2 and cytokine expression and reduce adhesion-related molecules (Hidalgo et al., 2012).

In summary, DHZCP may regulate vascular inflammation through multiple pathways, including NF-κB, TLR4-related signaling, and inflammasome-associated processes, with additional contributions from antithrombotic metabolites. Yet, the certainty of evidence is limited by heterogeneity of experimental designs, incomplete reporting of dosing and controls, and limited PAD-relevant causal validation.

Evidence level and limitations: Evidence was mainly derived from *in vitro*/*in vivo* inflammation or AS models; translation to PAD-specific vascular inflammation and clinical outcomes requires further validation. Key reporting gaps in the primary literature included incomplete dose/concentration ranges, minimal active exposure, limited use/reporting of positive/negative controls, and inconsistent characterization of intervention materials (commercial product vs. extract/serum), which reduced the rigor of mechanistic inference. Dose-response relationships and minimal active exposures were rarely established for anti-inflammatory readouts, and causal attribution to specific metabolites/pathways was limited by scarce inhibitor/genetic validation.

What the evidence shows/does not show: Reductions in inflammatory mediators and NF-κB-related signals were commonly reported, but whether these changes are necessary/sufficient to improve PAD outcomes was not demonstrated due to limited causal designs and PAD-specific validation. Incomplete reporting of controls, dosing, and material characterization further reduced certainty and should be addressed in future studies. In addition, most network-predicted inflammatory targets/pathways lacked PAD-relevant functional validation using inhibitors/agonists or genetic perturbation, limiting causal interpretation.

### 4.5 Oxidative stress and antioxidant defenses

Oxidative stress is widely recognized as a driver of AS and its peripheral manifestation, PAD. It is considered an initiator of vascular endothelial injury (Wai et al., 2021). Excessive ROS production damages endothelial cell structures, induces adhesion molecules such as ICAM-1 and VCAM-1, and promotes inflammatory mediator release, collectively contributing to endothelial barrier disruption, smooth muscle cell migration, intimal thickening, and plaque formation (Yan et al., 2024). Both clinical and experimental studies have suggested that oxidative stress plays a persistent role across the course of PAD, from early endothelial injury to later plaque destabilization (Signorelli et al., 2019). Accordingly, antioxidant-associated mechanisms are frequently discussed as an additional rationale for DHZCP in PAD-related vascular injury.

Modern pharmacological evidence suggests that DHZCP may modulate oxidative stress via multiple pathways. DHZCP was reported to reduce ROS generation, enhance endogenous antioxidant capacity, and inhibit oxidative stress-induced endothelial inflammation, thereby potentially supporting endothelial homeostasis (Fu C. et al., 2023). DHZCP was reported to increase antioxidant enzyme levels (SOD, CAT) and reduce oxidative injury in specific experimental contexts (Fu Y. et al., 2023). In an ox-LDL-induced endothelial injury model, DHZCP-containing serum was reported to influence ubiquitin-proteasome activity and NF-κB-related signaling, thereby attenuating endothelial dysfunction (Shi et al., 2025). Animal-derived materials likewise yielded antioxidant-related signals in some studies; for example, protein/peptide extracts from Qicong were reported to increase SOD/CAT and reduce ROS/MDA, possibly via Nrf2/HO-1 activation (Chen et al., 2024).

Taken together, available evidence suggests that DHZCP may alleviate oxidative stress-related endothelial damage by modulating PI3K/Akt/FoxO4-related signaling, reducing ROS, and supporting antioxidant defenses. However, many studies lacked detailed reporting of exposure/dosing or used models not specific to PAD, and causal links between these pathways and PAD outcomes remain to be established.

Evidence level and limitations: Most evidence was derived from *in vitro* endothelial injury models and non-PAD *in vivo* settings; therefore, the mechanistic conclusions should be regarded as suggestive until validated in PAD-relevant models with dose-exposure assessment. Key reporting gaps in the primary literature included incomplete dose/concentration ranges, minimal active exposure, limited use/reporting of positive/negative controls, and inconsistent characterization of intervention materials (commercial product vs. extract/serum), which reduced the rigor of mechanistic inference. Dose-response relationships and minimal active exposures were rarely established for antioxidant readouts, and the linkage between *in vitro* concentrations and *in vivo* achievable exposure in PAD-relevant settings remained unclear.

What the evidence shows/does not show: Antioxidant-associated readouts were reported *in vitro* and in non-PAD settings, but causal links between oxidative-stress modulation and PAD clinical improvement were not established. Non-physiological concentrations and limited pathway-blockade verification suggest that exposure-aware and causally informative PAD models are required.

### 4.6 Hemorheology, coagulation/platelet function, and microcirculation

Hemorheological abnormalities contribute to PAD progression, including increased blood viscosity, reduced erythrocyte deformability, enhanced platelet activation, and impaired microcirculatory perfusion, which aggravate distal ischemia and vascular occlusion (Valeanu et al., 2021). Since PAD progression and acute limb events involve both chronic atherosclerotic narrowing and thrombosis-prone blood flow conditions, hemorheological modulation is often discussed as a clinically relevant mechanistic domain. Modern research suggests that DHZCP may exert multi-target regulatory effects that improve hemorheological indices and local hemodynamics (Shi et al., 2025).

Pharmacological studies reported that DHZCP modulates coagulation and platelet activity, thereby potentially improving hemorheological status (Shi et al., 2025). Clinical and preclinical evidence suggests that plasma viscosity is associated with fibrinogen in atherosclerotic disease; DHZCP was reported to lower plasma viscosity, which may reduce the viscosity burden associated with fibrinogen (Poredos and Zizek, 1996). Regarding coagulation regulation, DHZCP was reported to inhibit thrombin activity and upstream factors such as FXIa, thereby potentially reducing coagulation amplification and thrombus formation (Shi et al., 2025). Several metabolites were suggested to affect platelet P2Y_1_/P2Y_12_-related signaling and TXA_2_/PGI_2_ balance, thereby reducing platelet aggregation.

Amygdalin from Taoren was reported to reduce plasma viscosity, prolong aPTT, and decrease fibrinogen in blood-stasis rat models (Liu et al., 2012). Xingren contains monounsaturated and polyunsaturated fatty acids, and long-term intake was reported to improve lipid profiles in broader nutritional literature, which may indirectly benefit microcirculation (Singar et al., 2024). Insect-derived medicinal materials such as Shuizhi, Zhechong, and Qicong contain anticoagulant peptides/proteins that were reported to inhibit thrombin/coagulation factors and reduce platelet aggregation, consistent with blood-activating principles (Kim et al., 2022; Chen et al., 2015; Xie et al., 2021). Nevertheless, heterogeneity in extracts/material definitions and incomplete reporting of dosing and bleeding-related safety endpoints limited firm clinical inference.

In summary, DHZCP may improve hemorheological abnormalities through anticoagulant, antiplatelet, and microcirculation-related mechanisms. However, the clinical relevance-especially in terms of hard PAD endpoints (e.g., restenosis, revascularization, amputation)-requires higher-quality studies with standardized outcome reporting and safety monitoring.

Evidence level and limitations: Evidence was derived from mixed preclinical models and limited clinical studies; dose-response relationships and bleeding risk assessments were seldom systematically reported. Key reporting gaps in the primary literature included incomplete dose/concentration ranges, minimal active exposure, limited use/reporting of positive/negative controls, and inconsistent characterization of intervention materials (commercial product vs. extract/serum), which reduced the rigor of mechanistic inference. Dose-response relationships and minimal active exposures were rarely established for hemorheological/antithrombotic effects, and bleeding-risk-relevant exposure thresholds were seldom evaluated systematically.

What the evidence shows/does not show: Signals consistent with improved hemorheology/antithrombosis were reported, but net clinical benefit-risk in PAD (especially bleeding risk under concomitant antiplatelet/anticoagulant therapy) was not established due to limited safety reporting and lack of hard endpoints. Future trials should prespecify bleeding outcomes and link rheological changes to functional and imaging-confirmed endpoints.

**FIGURE 2:**
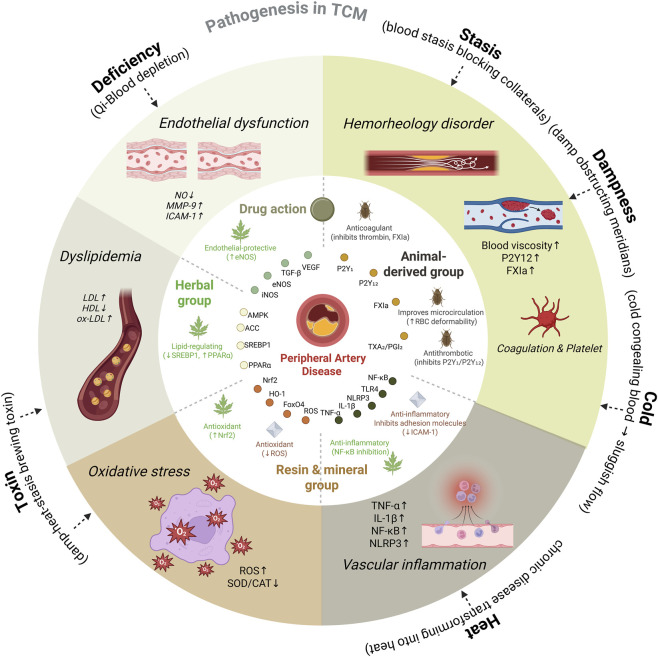
Conceptual framework linking TCM pattern/pathogenesis of peripheral artery disease (PAD) (*Tuoju*-related syndromes) with the putative multi-target and multi-pathway actions of DHZCP (evidence mapped across *in silico*, *in vitro*, *in vivo*, and clinical studies).

DHZCP exhibits a multi-target, multi-pathway intervention profile in the treatment of PAD (Figure 2). The coordinated actions of both botanical and insect-derived medicines may regulate key pathological processes, including endothelial dysfunction, lipid metabolism imbalance, inflammation, oxidative stress, and hemorheological abnormalities. Together, these mechanisms provide a plausible mechanistic rationale for its potential application in peripheral atherosclerotic vascular diseases. Nevertheless, much of the current mechanistic evidence is indirect (non-PAD models) and should be regarded as suggestive until validated with PAD-relevant models, exposure-aware dosing, and causal experiments.

Overall evidence boundary: Across mechanistic domains, the literature supports biological plausibility based mainly on surrogate readouts, but definitive mechanisms and PAD-specific functional validation remain major gaps.

## 5 Clinical evidence and safety

### 5.1 Clinical evidence overview and certainty of evidence

As mechanistic hypotheses for DHZCP have expanded—covering lipid regulation, anti-inflammatory action, antithrombotic effects, and improvements in endothelial functions—its potential clinical application in PAD has garnered increasing attention. Existing clinical studies consist primarily of small randomized controlled trials (RCTs), controlled observational studies, and combination regimens that integrate DHZCP with conventional therapies and/or other TCM interventions (Table 2 (Lu, 2019; Shi et al., 2021; Yu et al., 2021; Chen et al., 2025)). These studies include both patients with PAD alone and those with diabetes complicated by PAD.

**TABLE 2:** Summary of clinical studies on Dahuang Zhechong pill (DHZCP) for lower extremity peripheral artery disease (PAD).

Study (author, year)	Design	Population	Intervention	Comparator	Key outcomes measured	Major findings (concise)	Key limitations/risk-of-bias signals
Yu et al. (2021)	RCT	Diabetes with lower-limb PAD (NR for diagnostic criteria)	Sun’s abdominal acupuncture + DHZCP + routine care	Cilostazol (± routine care; NR)	FPG, HbA1c, TC, TG, LDL-C, IMT, PWV, ABI, response rate	Reported improvements in metabolic indices; reduced IMT/PWV; increased ABI vs. comparator	Randomization/blinding details NR; sample size NR; co-interventions present (acupuncture); follow-up duration NR; Flag: poor preparation reporting (product specification/dose NR); AE/bleeding monitoring NR
Lu (2019)	Prospective controlled study (post-intervention PAD)	Post-endovascular PAD (NR)	Routine care + DHZCP + modified Xuefu Zhuyu Decoction (NR for dose/duration)	Routine care	Lipids (TC, TG, LDL-C, HDL-C), fibrinogen, hemorheology, ABI, restenosis rate	Reported better lipid/hemorheology/ABI outcomes and lower restenosis signal	Non-randomized; co-intervention (another formula) confounds attribution; restenosis assessment method NR; Flag: poor preparation reporting (product specification/dose/duration/batch-manufacturer information NR); AE reporting limited/NR
Chen et al. (2025)	Controlled clinical study (NR)	PAD or PAD-related population (NR)	DHZCP + modified Xuefu Zhuyu Decoction (NR for dose/duration)	Routine care (NR)	Lipids (TC, TG, LDL-C, HDL-C), fibrinogen, hemorheology, ABI, restenosis rate	Reported improved lipids/fibrinogen/coagulation and increased ABI/walking capacity	Allocation method NR; blinding NR; sample size/follow-up NR; outcome definitions NR; Flag: poor preparation reporting (product specification/dose/duration/batch-manufacturer information NR); AE/bleeding risk monitoring NR
Shi et al. (2021)	Controlled clinical study (NR)	PAD (NR)	DHZCP + routine care	Routine care (NR)	Lipids, fibrinogen, coagulation indices, ABI, pain-free walking distance	Reported improved lipids/hemorheology and ABI; possible restenosis reduction	Potential overlap with other reports-needs verification; design/registration NR; co-interventions possible; Flag: poor preparation reporting (product specification/dose/duration NR); AE reporting NR

“NR” indicates not reported in the primary study report. Studies flagged as having poor preparation reporting had insufficient information on product specification, dose, duration, and/or batch-manufacturer details. Across the included clinical studies, it was generally unclear whether the exact DHZCP, preparations were identical or directly comparable.

Across the existing literature, efficacy was most commonly assessed using surrogate or intermediate endpoints, including the ankle-brachial index (ABI), pulse wave velocity (PWV), and Doppler-based hemodynamic parameters (Zhang Y et al., 2025). Overall, DHZCP exposure was reported to be associated with increases in ABI, reductions in PWV, and improvements in peripheral blood-flow dynamics. With respect to cardiometabolic risk factors, DHZCP was repeatedly reported to reduce TC, TG, and LDL-C while increasing HDL-C (Shi et al., 2025). Several studies also reported changes in endothelial-related biomarkers (e.g., NO and ET-1) (Fu C. et al., 2023), and improvements in hemorheological/coagulation-related indices (e.g., plasma viscosity and fibrinogen). Moreover, reductions in inflammation-associated biomarkers (hs-CRP and Hcy) were observed in some studies (Shang and Li, 2023). Taken together, these findings provide preliminary clinical signals that are directionally consistent with the mechanistic hypotheses summarized above; however, the clinical certainty remains limited.

Critical appraisal (clinical evidence quality): Most clinical studies were small, short-term, and heterogeneous in co-interventions, with limited reporting of randomization/blinding and reliance on surrogate endpoints (ABI, PWV, biomarkers) rather than hard outcomes (amputation-free survival, restenosis confirmed by imaging, revascularization, MACE). In addition, detailed reporting of DHZCP product specifications, dosing regimens, follow-up duration, and adverse-event monitoring (particularly bleeding risk when combined with antiplatelet/anticoagulant therapy) was frequently incomplete, which limits confidence in effect attribution and benefit-risk assessment. Notably, the limited number of eligible clinical studies reflects the current evidence landscape rather than selective inclusion, underscoring the need for additional well-designed trials.

In summary, although the current evidence is derived mainly from small-sample RCTs and observational studies, DHZCP may provide incremental benefit as an adjunct to conventional therapy in PAD and diabetes-complicated PAD. Reported benefits include improved lower-limb perfusion, partial lipid modulation, favorable changes in endothelial biomarkers, and mitigation of hypercoagulability-related indices. Nevertheless, long-term efficacy and safety require confirmation in larger, preregistered, multicenter trials with standardized background therapy, predefined hard endpoints, and rigorous safety monitoring.

### 5.2 Monotherapy and adjunct use: reported outcomes and boundaries

Existing clinical and preclinical studies have preliminarily evaluated the efficacy of DHZCP in PAD and related atherosclerotic conditions using multidimensional endpoints, including vascular function, lipid metabolism, hemorheology, and inflammatory markers (Song and Yue, 2024). Among these, vascular functional indices have been a major focus. Studies reported that DHZCP was associated with increased ABI and reduced PWV, suggesting potential improvements in arterial compliance and lower-limb perfusion efficiency.

In terms of lipid metabolism, DHZCP was reported to show a lipid-modulating effect in some studies (Huang and Shen, 1989). These findings are directionally consistent with the proposed rationale of attenuating lipid accumulation and slowing atherosclerotic progression. Regarding hemorheology, evidence from related disease contexts suggests that DHZCP may reduce whole-blood viscosity, plasma viscosity, and fibrinogen levels, thereby improving blood fluidity and reducing risks for thrombosis (Shi et al., 2025). However, these data are often indirect and not specific to PAD populations.

Evidence boundary: Monotherapy evidence mainly supports short-term changes in surrogate outcomes, while comparative effectiveness versus guideline-directed therapy and impact on hard PAD outcomes remain unproven.

In summary, available studies suggest that DHZCP may influence vascular function, lipid indicators, and hemorheological/inflammatory readouts. However, given limitations in study design, sample size, and endpoint selection, these findings should be interpreted as preliminary and hypothesis-supporting rather than definitive evidence of monotherapy efficacy. Large-scale, long-term RCTs are still needed to substantiate clinical benefit and safety.

### 5.3 Combination strategies and attribution of incremental benefit

Current clinical evidence suggests that DHZCP may yield complementary benefits when used in combination therapy, including integration with acupuncture, formula modification based on syndrome differentiation, and combination with conventional medications such as cilostazol (Guo and Sun, 2025). Conceptually, these approaches aim to address both systemic risk factors and local perfusion impairment through multi-domain regulation; however, they also introduce co-intervention confounding that must be considered when interpreting efficacy claims.

In acupuncture-herbal combination regimens, DHZCP was frequently administered alongside meridian-based point stimulation intended to enhance lower-limb blood flow, potentially improving perfusion and supporting ischemic tissue recovery (Sun et al., 2025). A RCT reported that abdominal acupuncture (Sun’s method) plus DHZCP, on top of conventional medication, improved ABI, carotid intima-media thickness (IMT), PWV, and limb hemodynamics compared with cilostazol-based control regimens (Yu et al., 2021). Notably, because acupuncture was part of the intervention package, the incremental effect attributable to DHZCP alone could not be isolated.

The “formula modification” model reflects syndrome differentiation and individualized treatment. Some studies adopted DHZCP as a base prescription and combined it with adjunct formulas such as Xuefu Zhuyu Decoction according to cold–heat and deficiency–excess patterns. These studies reported larger improvements in TG, LDL-C, and fibrinogen compared with unmodified regimens (Guo and Sun, 2025). However, interpretability is limited unless the control arm is matched for background therapy and co-interventions.

In integrative TCM–Western medicine regimens, DHZCP has been combined with cilostazol. Given cilostazol’s antiplatelet and hemorheological actions, the combination may provide broader coverage across perfusion, endothelial-related biomarkers, and inflammatory/thrombotic indices (Chen et al., 2025). Nevertheless, additive benefit should ideally be confirmed using rigorously controlled designs with standardized co-intervention protocols.

**FIGURE 3:**
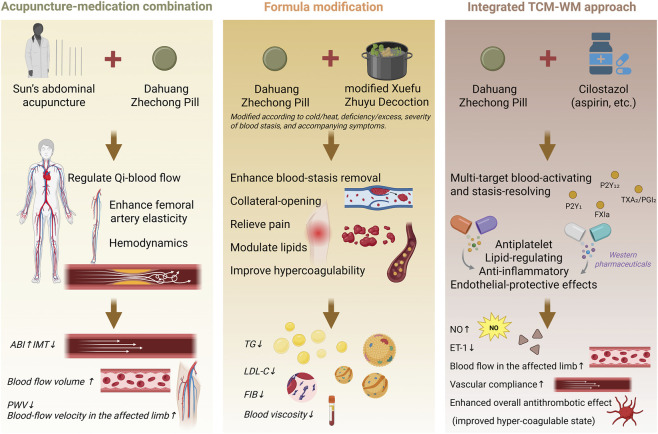
Schematic overview of three commonly reported combination-therapy models involving DHZCP in PAD management: (i) DHZCP plus acupuncture, (ii) DHZCP-based formula modification (syndrome differentiation), and (iii) DHZCP combined with conventional pharmacotherapy.

In summary, whether in acupuncture-herbal, modified formula, and integrative therapy models, DHZCP was consistently reported to be associated with improvements in surrogate vascular and hemorheological outcomes. At the same time, the complexity of co-interventions highlights the need for trial designs that can quantify incremental benefit and clarify attribution. These findings support the potential value of multi-metabolite formula-based strategies for multifactorial PAD (Figure 3).

### 5.4 Safety and tolerability

Current clinical studies commonly suggest that DHZCP has acceptable short-term tolerability in PAD populations (Lv et al., 2021). Most studies did not report any serious adverse events, and the reported adverse reactions were generally mild and reversible (Ye et al., 2022). Common symptoms included transient gastrointestinal discomfort (e.g., mild diarrhea or abdominal distension) and nonspecific symptoms such as dry mouth and fatigue. Given that DHZCP contains Dahuang (a purgative botanical drug), increased bowel movements may occur in some patients; therefore, individualized dose adjustment and close monitoring are recommended in routine practice.

Across observational and prospective studies, DHZCP, used alone or in combination, was generally reported not to cause significant abnormalities in routine blood tests, liver/kidney function, or coagulation parameters. Some studies in non-PAD indications reported improvements in hepatic function, whereas elevated transaminase levels were reported in a small number of cases when combined with specific drugs (e.g., adefovir) (Dai et al., 2021). In studies of diabetes complicated by PAD, DHZCP was often co-administered with acupuncture or other TCM formulas, and no increase in serious adverse events was reported compared with cilostazol-based regimens. However, systematic bleeding-risk assessment and drug–drug interaction reporting were often insufficiently detailed, which limits firm conclusions on long-term safety in patients receiving antiplatelet/anticoagulant therapy.

As an oral Chinese patent medicine, DHZCP is convenient to administer. In studies of other chronic conditions (e.g., pneumoconiosis, hepatic fibrosis, cancer), treatment duration often ranged from weeks to months with low dropout rates, suggesting reasonable adherence. Symptom improvements such as increased claudication distance, reduced limb pain, and increased peripheral temperature were reported in some studies, which may support adherence (Ye et al., 2022). Nevertheless, these observations are indirect for PAD and should not be over-interpreted as the evidence of PAD-specific efficacy.

In summary, available clinical evidence suggests a generally favorable short-term safety profile for DHZCP in PAD; however, confirmation of long-term safety and the benefit-risk profile requires large-scale, multicenter RCTs with standardized adverse-event reporting, including bleeding outcomes and drug-drug interaction monitoring, to meet international evidence standards.

## 6 Conclusions and future directions

### 6.1 Key takeaways: mechanistic plausibility vs. clinical certainty

Current evidence suggests that DHZCP has been associated with a multi-domain mechanistic profile that is directionally consistent with key pathological processes in PAD, including endothelial dysfunction, lipid deposition, chronic inflammation, oxidative stress, and hemorheological abnormalities. Multiple bioactive plant metabolites and animal-derived bioactive substances in the formula have been linked to targets such as VEGF, eNOS, MMP-9, NF-κB, ICAM-1, PAF, and P2Y receptors, reflecting a broad regulatory profile (Shi et al., 2025). However, much of the mechanistic evidence was generated in non-PAD experimental settings, and many pathways were supported by associative biomarker readouts rather than causally informative designs; therefore, the mechanistic literature should be interpreted as biologically plausible rather than definitive.

Evidence gaps: causality, dose-response, and PAD-specific validation. First, the most frequently discussed candidate bioactive plant metabolites potentially contributing to DHZCP effects included emodin, rhein, baicalin/baicalein, paeoniflorin, and catalpol; however, none can currently be designated as definitive “core actives” for PAD due to limited exposure-aware and causally informative evidence. Second, clear dose-response relationships were seldom established because minimal active concentrations, tested dose ranges, and pharmacokinetic exposure data linking *in vitro* activity to *in vivo* achievable levels were often not reported. Third, although network approaches and molecular docking proposed multiple targets/pathways, functional validation in PAD-relevant limb ischemia/arterial stenosis models using inhibitors/agonists or genetic perturbation (knockdown/knockout) remained scarce, limiting causal interpretation. Consequently, in this review, *in silico* predictions were treated as hypothesis-generating signals rather than mechanistic proof.

From a mechanistic perspective, DHZCP has been associated with changes in multiple signaling pathways across experimental studies. For example, anti-inflammatory and antioxidant signals have been linked to p38 MAPK/NF-κB pathways in selected experimental models (Fu Y. et al., 2023). Meanwhile, experimental and network-based studies have suggested potential effects on coagulation factor and platelet-related signaling, providing a mechanistic rationale for antithrombotic and hemorheology-related signals (Shi et al., 2024). Moreover, several classes of constituents—such as flavonoids from Huangqin, fatty acids from Taoren, saponins from Gancao, and animal-derived bioactive peptides/proteins—have been implicated in synergistic anti-inflammatory, antioxidant, and microcirculatory effects. Nevertheless, the proposed systems-level or network-based rationale should be regarded as a conceptual framework unless key predicted targets are confirmed using exposure-aware and causally informative experiments in PAD-relevant models (Wang et al., 2025; Lien et al., 2017; Huang et al., 2019; Yu et al., 2022).

Comparison with conventional pharmacotherapies further underscores this conceptual difference. Statins primarily target HMG-CoA reductase to modulate lipid metabolism, while antiplatelet agents often act on COX or P2Y_12_ receptors. In contrast, DHZCP has been associated with concurrent modulatory effects on lipid profiles, endothelial biomarkers, inflammatory mediators, oxidative stress indices, and hemorheological parameters. Nevertheless, mechanistic breadth alone does not translate into superior clinical benefit. Demonstration of comparative effectiveness requires adequately powered trials incorporating standardized background therapy and clinically meaningful hard endpoints.

Future mechanistic studies should aim to refine the formula’s “metabolite-target-pathway” network through multi-omics approaches by identifying bioactive metabolites, clarifying *in vivo* metabolism/exposure, validating pathways experimentally, and applying genetic or pharmacological perturbation strategies. Such work is essential to move from associative biomarker changes toward causal inference and clinically translatable mechanism clarification.

### 6.2 Clinical application prospects and translational considerations

With the rising prevalence of PAD—particularly among individuals with diabetes and older adults—conventional therapies (antiplatelet agents, statins, vasodilators, and revascularization procedures) can improve symptoms and perfusion but remain limited by incomplete risk-factor control, restenosis, adherence challenges, and cumulative adverse effects. Against this background, DHZCP has been explored as an adjunct option, given its multi-domain regulatory signals spanning vascular inflammation, endothelial function, thrombosis-prone hemorheology, and metabolic risk factors.

Clinical studies have reported associations between DHZCP use and improvements in lower-limb perfusion (e.g., ABI), lipid indicators, fibrinogen-related indices, and hemorheological parameters. Several studies also reported changes in endothelial-related biomarkers, suggesting potential endothelial benefits. Compared with Western medications alone, the formula has been proposed to offer broader coverage across interacting pathological domains. Nevertheless, these prospects should be interpreted cautiously because current clinical evidence is largely based on small, short-term studies with heterogeneous co-interventions and limited hard endpoints.

In chronic disease management, DHZCP may be considered an adjunct during early-stage vascular dysfunction or during post-intervention recovery to support hemodynamic stabilization and potentially reduce restenosis risk. Preliminary evidence suggests potential synergy when combined with agents such as cilostazol or aspirin, although benefit attribution and safety (especially bleeding risk under concomitant antithrombotic therapy) require more rigorous confirmation. Looking ahead, as identification of key bioactive metabolites, pharmacokinetic characteristics, and therapeutic networks progresses, DHZCP could play a more clearly defined role within an evidence-based framework for PAD. However, translation to guideline recommendations will depend on multicenter trials with long-term follow-up and standardized outcome reporting.

### 6.3 Limitations

Despite promising signals, the current evidence base for DHZCP in PAD has several important limitations. First, mechanistic evidence is frequently indirect, as many studies used non-PAD models (e.g., generic AS or endothelial injury models), which limits translatability to lower-limb perfusion and limb-event outcomes. Second, the plausibility of dose-exposure relationships is often unclear since concentration ranges, minimal active concentrations, and pharmacokinetic exposure data were incompletely reported, particularly in *in vitro* studies and serum pharmacology. Third, intervention materials were heterogeneous (commercial products, laboratory decoctions/extracts, serum pharmacology, or isolated metabolites), while product specifications (batch number, manufacturer, certificate of analysis) and material characterization were frequently missing, reducing reproducibility and cross-study comparability. Fourth, clinical studies were commonly small, short-term, and heterogeneous in co-interventions, with limited reporting of randomization/blinding and reliance on surrogate endpoints (ABI, PWV, biomarkers) rather than hard outcomes (amputation-free survival, imaging-confirmed restenosis, revascularization, MACE). Finally, safety reporting—especially bleeding risk and drug-drug interactions when combined with antiplatelet/anticoagulant therapy—was often insufficient for firm benefit–risk assessment.

### 6.4 Future research needs and priorities

Although current studies have preliminarily suggested multi-target therapeutic potential of DHZCP in PAD, the overall evidence remains insufficient. First, large-scale, multicenter RCTs should be conducted using standardized diagnostic criteria, prespecified outcomes, and appropriate follow-up. In particular, inclusion of hard endpoints—such as amputation rates, major adverse cardiovascular events, and mortality—would strengthen generalizability and international credibility.

Second, mechanistic studies should adopt an integrated “formulation-metabolite-target-pathway-effect” framework. Future investigations should focus on identifying key bioactive metabolites, elucidating *in vivo* metabolic behavior and exposure, and clarifying target-specific regulatory mechanisms. High-resolution pharmacological networks may be constructed using transcriptomics, metabolomics, and single-cell sequencing technologies. Moreover, experimental systems—including *in vitro* models, PAD-relevant ischemia/arterial stenosis animal models, and clinical biomarkers—should be triangulated to explore causal links between pharmacodynamic effects and clinical endpoints (e.g., NO/ET-1 homeostasis, inflammatory cytokine profiles, perfusion/hemodynamic parameters).

Third, high-quality evidence supporting integrative TCM-Western medicine strategies remains insufficient and should be strengthened. Future investigations should evaluate differentiated therapeutic frameworks, such as DHZCP-based therapy combined with guideline-directed medical therapy or adjunctive use alongside endovascular or surgical revascularization, in order to delineate optimal timing and quantify incremental benefit across perioperative, rehabilitation, and chronic ischemic phases. Moreover, the potential for pharmacodynamic synergy, add-on effects, or therapeutic substitution in combination with statins, antiplatelet agents, and vasodilators warrants systematic and rigorously designed evaluation.

In addition, safety evaluation and pharmacovigilance should be expanded, with particular attention to hepatic/renal function, bleeding risk, and drug-drug interactions in elderly patients and those with multimorbidity. Finally, regarding standardization and quality control, further work is recommended to clarify bioactive metabolites and quality markers of the formula—including animal-derived ingredients—to facilitate a modern, reproducible, and regulatorily compliant pharmaceutical framework.

Priority research agenda (summary): (1) PAD-relevant *in vivo* models with exposure-aware dosing and causal validation (inhibitors/genetic perturbation); (2) preregistered, adequately powered multicenter trials with hard endpoints and prespecified bleeding/drug–drug interaction monitoring; and (3) standardized DHZCP material characterization (including insect-derived drugs) and batch consistency reporting to improve reproducibility.

In this review, network approaches and molecular docking results were treated as hypothesis-generating signals rather than mechanistic evidence, and conclusions were primarily based on experimentally validated *in vitro/in vivo* findings and available clinical data. Overall, future progress will require coordinated advances in mechanistic causality, clinical evidence strength, and standardization/quality control to establish a robust scientific basis for DHZCP in PAD.
